# MiRNA sequencing of platelet and exosome revealed platelet miR-199b-3p as a potential biomarker in lung adenocarcinoma

**DOI:** 10.3389/fimmu.2025.1619448

**Published:** 2025-08-29

**Authors:** Xiaoxia Wen, Sisi Yu, Ping Leng, Yu Liu, Huaichao Luo

**Affiliations:** ^1^ Department of Clinical Laboratory, Sichuan Clinical Research Center for Cancer, Sichuan Cancer Hospital & Institute, Sichuan Cancer Center, University of Electronic Science and Technology of China, Chengdu, China; ^2^ Chongqing Key Laboratory of Sichuan-Chongqing Co-Construction for Diagnosisand, Treatment of Infectious Diseases Integrated Traditional Chinese and Western Medicine, College of Medical Technology, Chengdu University of Traditional Chinese Medicine, Chengdu, Sichuan, China; ^3^ Department of Medical Oncology, Sichuan Cancer Hospital and Institute, Chengdu, Sichuan, China; ^4^ Transfusion Medicine Research Center, The Institute of Blood Transfusion, Chinese Academy of Medical Sciences and Peking Union Medical College, Chengdu, China

**Keywords:** platelet, exosome, miRNA sequencing, lung adenocarcinoma, reference miRNA, liquid biopsy, hsa-miR-199b-3p

## Abstract

**Background:**

Distinguishing malignant lung adenocarcinoma (LAC) from benign pulmonary nodules (BPN) is a major clinical challenge. While exosomal microRNAs (miRNAs) are established liquid biopsy biomarkers, tumor-educated platelet miRNAs represent an emerging source. However, a direct comparison of the diagnostic potential between these two sources, and the identification of reliable platelet miRNA biomarkers for LAC, remain poorly defined. Critically, the well-documented interaction between tumor-derived exosomes and platelets, which complicates exosome isolation due to inevitable platelet contamination, raises a pivotal question: Could the analysis of platelet miRNA, a far easier-to-isolate component, offer a viable and efficient alternative to exosome-based diagnostics?

**Methods:**

We performed miRNA sequencing (miRNA-seq) on paired peripheral blood platelets and plasma exosomes from healthy donors (HD), BPN, and LAC patients. The abundance and diversity of miRNAs were compared. Candidate reference miRNAs for platelet studies were screened and validated using RT-qPCR and multiple stability algorithms. Differentially expressed platelet miRNAs were identified and validated in a cohort of 133 subjects (70 LAC, 31 HD, 32 BPN). The diagnostic performance of the top candidate was evaluated using ROC analysis and Net Reclassification Index (NRI) against traditional biomarkers (CEA) and clinical models. Target genes were predicted using bioinformatic tools and validated with public databases (TCGA, UALCAN, GEPIA).

**Results:**

Platelets contained significantly greater miRNA diversity and abundance compared to exosomes. Differentially expressed platelet miRNAs showed higher concordance with tissue-specific signatures than their exosomal counterparts. hsa-let-7i-5p was identified as the most stable reference miRNA for normalizing platelet miRNA expression in LAC. hsa-miR-199b-3p was significantly downregulated in the platelets of LAC patients compared to both HD and, crucially, BPN patients. It effectively distinguished LAC from BPN (AUC = 0.73) and early-stage LAC (Stage I) from BPN (AUC = 0.72), outperforming traditional biomarkers (CEA) and clinical models, as confirmed by significant NRI values. The diagnostic value of miR-199b-3p was also significant in the non-GGN subgroup (p=0.037). Bioinformatic analysis predicted KTN1 as a key target gene, with an inverse correlation to miR-199b-3p in LAC tissues and association with poor prognosis. Intriguingly, KTN1 expression in platelets was also dysregulated, suggesting a complex platelet-tumor interaction.

**Conclusion:**

This study demonstrates that platelet miRNAs are a superior source for liquid biopsy in LAC compared to exosomal miRNAs. We establish hsa-let-7i-5p as a reliable reference gene and identify platelet hsa-miR-199b-3p as a promising non-invasive biomarker for the differential diagnosis of malignant and benign pulmonary nodules, offering a new avenue for the early detection of lung adenocarcinoma.

## Highlights

- Sequencing results revealed that platelets exhibit greater diversity and abundance of miRNAs compared to exosomes.
- Differentially expressed platelet miRNAs demonstrate higher concordance with tissue-specific miRNAs, underscoring their potential as liquid biopsy biomarkers.
- The let-7i-5p miRNA is identified as a suitable reference miRNA for platelet studies in lung adenocarcinoma research.
- Platelet hsa-miR-199b-3p shows promise as a potential biomarker for distinguishing lung adenocarcinoma from benign pulmonary nodules.

## 1 Introduction

Lung cancer is the leading cause of cancer-related deaths and the second most diagnosed malignancy worldwide and Lung adenocarcinoma (LAC) is a highly heterogeneous tumor and the most prevalent pathological type of lung cancer (1). In recent years, there has been a significant increase in research focusing on the occurrence, progression, and treatment of LAC (2–5). Advancements in diagnosis, surgical techniques, radiotherapy, immunotherapy, and molecular-targeted therapies have led to significant improvements in clinical outcomes for LAC patients (6). However, the 5-year survival rate for LAC patients remains low (7). Studies have demonstrated that aggressive surgical intervention can significantly improve prognosis for patients with early-stage LAC (8). However, early-stage diagnosis of LAC remains a significant clinical challenge due to the absence of specific symptoms and the similarity of CT manifestations to those of benign pulmonary nodules (9). Some biomarkers such as carcinoembryonic antigen (CEA), and neuron-specific enolase (NSE) were used to diagnose lung cancer while the diagnostic abilities of these serum markers are limited in sensitivity and specificity (10–13). Therefore, there is an urgent need for novel biomarkers capable of reliably differentiating between benign and malignant lung nodules.

MiRNAs are a class of small non-coding RNAs, typically 19–25 nucleotides in length, that post-transcriptionally regulate gene expression (14, 15). Cancer is a complex genetic disease caused by the accumulation of mutations, which lead to deregulation of gene expression and uncontrolled cell proliferation (16). MiRNAs have been shown to play important roles in carcinogenesis (17). MiRNAs are widely found in tissues, cells, serum, plasma, and exosomes (18). Research has demonstrated that many of the biological effects of exosomes can be attributed to MiRNAs. Furthermore, MiRNAs within exosomes are considered to have a potential role as biomarkers for cancer diagnosis (18, 19).

Platelets are the second most abundant cells in circulating peripheral blood produced, which play an important role in hemostasis and wound healing (20). The platelet counts, and platelet sizes contained a lot of clinical information (21). More importantly, the relationship between platelets and cancer has been studied since the last century (22, 23). Platelets are rich in RNA, including messenger RNA (mRNA), transfer RNA (tRNA), ribosomal RNA (rRNA), small nucleolar RNA (snoRNA), and miRNA (24). Platelet RNA has several advantages as biomarkers for cancer diagnosis, including high abundance, ease of isolation, relatively high RNA quality, and the ability to respond to external signals for RNA processing (23). Additionally, platelet miRNAs have been proven to serve as a blood-based biosource for liquid biopsies and have become a research hotspot for their potential diagnostic value in the early detection of tumors (25, 26). Therefore, platelets may serve as potential biomarker for a diagnosis of LAC.

Platelets, exosomes, and tumors share an intricate relationship. Two particularly direct lines of evidence demonstrate this connection. First, tumor-derived CD63-positive exosomes enter platelets leading to platelet activation, which further promotes tumor progression (27). Second, integrin dimers on tumor exosomes derived from lung interacts with platelet glycoprotein Ib, promoting platelet aggregation (28).

Critically, this very interaction between exosomes and platelets—central to tumor progression—complicates diagnostic approaches. While exosome-based liquid biopsy for microRNA (miRNA) analysis is a well-established approach for tumor diagnosis (29, 30), its clinical utility is hampered by significant challenges in exosome isolation. Compounding this difficulty is the recognized issue of platelet contamination (31). Consequently, leading experts recommend platelet depletion as a critical step prior to exosome purification (31, 32). This raises a pivotal clinical question: Given the technical complexity of exosome extraction and the evidence suggesting that some miRNA signal may derive from platelets – a component far easier to isolate – could direct analysis of platelet-derived miRNA offer a viable and efficient alternative (27)? To definitively address this question and assess the relative diagnostic value of these sources, a rigorous, head-to-head comparative analysis between platelet-depleted exosomes and the miRNA content of the platelets removed during depletion is imperative. Such a study is essential to determine whether platelet miRNA profiling could simplify and improve liquid biopsy diagnostics.

Here, we performed miRNA sequencing (miRNA-seq) on isolated peripheral blood platelets and plasma exosomes to explore the differences in miRNA expression between platelets and exosomes, and to investigate the relationship between platelet miRNA expression and LAC. We also screened four candidate reference miRNAs and verified their stability. Differentially expressed platelet miRNAs were selected and validated to demonstrate their potential value in distinguishing LAC patients from healthy donors (HD) and benign pulmonary nodules (BPN) patients. This provides new directions for the precise and early diagnosis of LAC.

## 2 Methods

### 2.1 Study participates

This study recruited participants from September 2021 to September 202 from Sichuan Cancer Hospital, comprising three groups: HD, BPN,and LAC. Inclusion criteria for BPN and LAC groups were (1): Patients with pulmonary nodules initially detected by low dose computed tomography (LDCT) who underwent surgery or histological examination at Sichuan Cancer Hospital. (2) For LAC group: Pathological diagnosis confirmed by two independent pathologists through NCCN clinical practice guidelines in oncology (Non-small cell lung cancer 2021); staging according to the 8th edition of the Union for International Cancer Control (UICC) TNM classification; no prior anti-neoplastic therapy; no history of treatments affecting platelet function (e.g., blood transfusion, aspirin); absence of other malignancies, infections, or inflammatory conditions; complete clinical information available. (3) For BPN group: Postoperative pathology confirming non-malignant status; no history of treatments affecting platelet function; absence of other malignancies, infections, or inflammatory conditions; complete clinical information available. (4) The HD group consisted of individuals with no evidence of LAC on follow-up low-dose spiral CT screening, and no other critical diseases, infections, or inflammatory conditions. This study was approved by the Medical Ethics Committee of Sichuan Cancer Hospital (approval number: SCCHEC-02-2020-043).

### 2.2 Platelet and plasma exosome isolation and purity assessment

**Figure 1:**
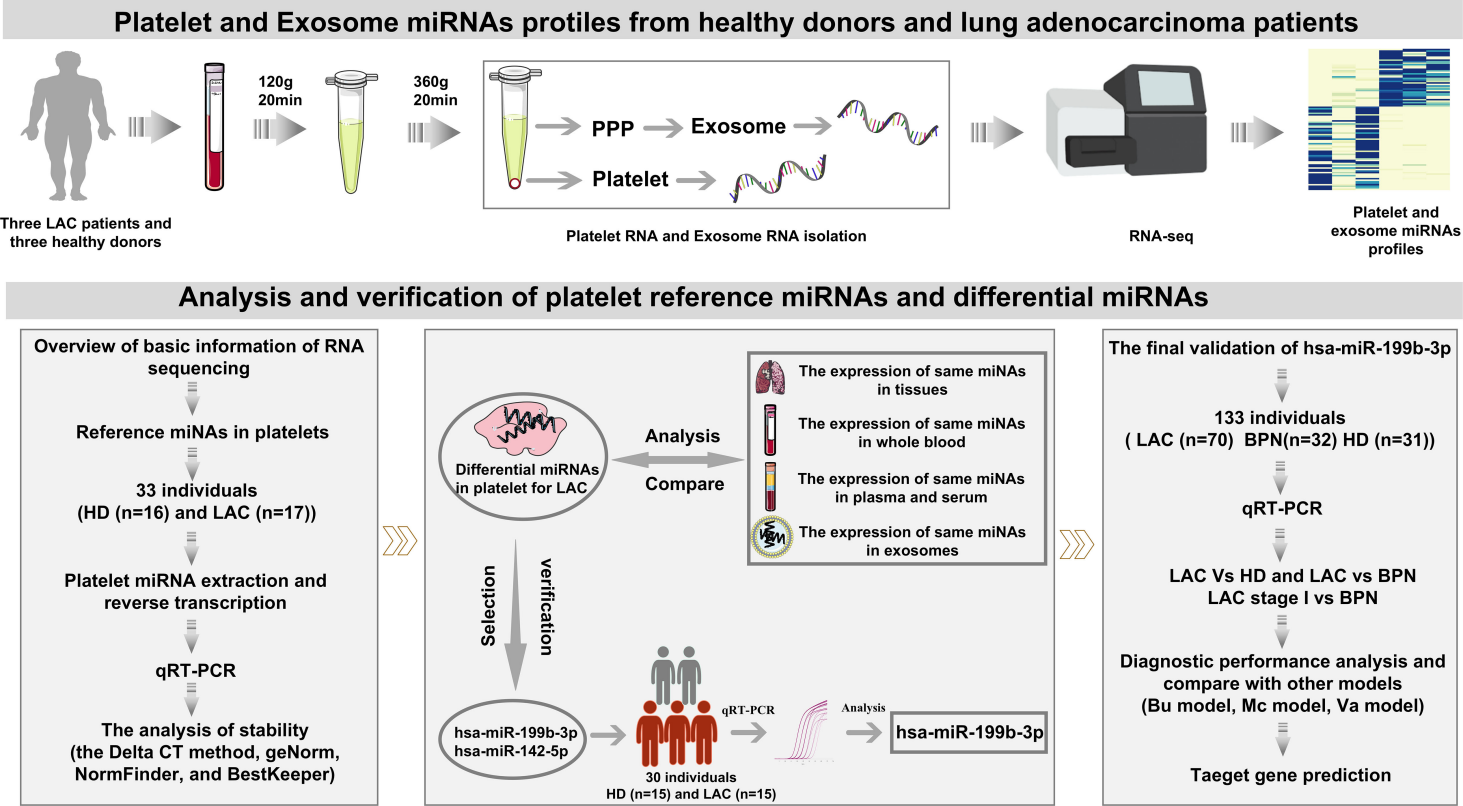
Schematic presentation of experimental workflow.

The work-flow chart of this study was shown in **Figure 1**. Platelets and plasma were isolated from whole blood collected in purple-cap BD Vacutainers containing EDTA anti-coagulant by standard centrifugation. The nucleated blood cells were removed by centrifugation at room temperature for 20 min at 120g (Co., Ltd. Shuke, Chengdu, China), resulting in platelet-rich plasma (PRP). To avoid leukocytes and erythrocytes contamination, only used the upper 4/5 of the PRP for further collecting platelets. The platelets were isolated from the PRP by centrifugation at room temperature for 20 min at 360g and the platelet pellet was collected in 50 μl RNAlater (Sigma-Aldrich, USA). Platelet purity was determined by morphometric experiments and platelet counting by the standard of less than five white blood cells per million platelets. In addition, the upper strata platelet-poor plasma (PPP) was stored directly at -80°C for further exosome isolation. To minimize platelet RNA degradation due to temporal effects, the isolation was supposed to be completed within 2h after blood collection.

The exosomes were isolated by SEC (size exclusion chromatography) methods as described previously with minor modifications (33). Plasma exosomes are mainly identified by Transmission electron microscopy (H-7650, Hitachi Ltd., Tokyo, Japan) and Nanoparticle Tracking Analysis. The identification results were consistent with the reported exosome particle size, and the morphological characteristics proved that the exosome extraction was qualified, (**Supplementary Figure 1**). All the samples were collected between 2021 and 2022.

### 2.3 Platelet and exosome RNA-sequencing

A total RNA rapid extraction kit for blood liquid sample (centrifugal column type) (Bioteke, China) were used to isolate platelet total RNA, following the manufacturer’s instructions. Exosome total RNA was extracted and purified from plasma exosome using miRNeasy Serum/Plasma Advanced Kit (Qiagen, cat. No. 217204) according to the kit instruction. And the assessments of the concentration and quality and purity of platelet and plasma RNA for sequencing were evaluated using the RNA Nano 6000 Assay Kit of the Agilent Bioanalyzer 2100 System (Agilent Technologies, CA, USA). Test reports of sequenced platelets and exosomes are shown in **Supplementary Table 1**.

The sequencing libraries were generated by QIAseq miRNA Library Kit (Qiagen, Frederick, MD) following manufacturer’s recommendations. Library quality was assessed by Agilent Bioanalyzer 2100 and qPCR. The clustering of the index-coded samples was performed on the acBot Cluster Generation System using TruSeq PE Cluster Kitv3-cBot-HS (Illumina, San Diego, CA, USA) according to the manufacturer’s instructions. After cluster generation, the library preparations were sequenced on an Illumina Hiseq platform and paired-end reads were generated.

### 2.4 Quantification analysis of miRNAs

The original image Data files sequenced by Illumina HiSeq2500 platform were converted into Raw Data by Base Calling, and the results were stored in FASTQ file format. It contains the information of sequencing sequence and its corresponding sequencing quality information. To ensure the accuracy of information analysis, quality control of the original data was required to obtain high-quality sequences (namely Clean Reads). The quality control criteria of the original sequences were as follows: (1) The sequence with low quality value was removed; (2) Reads with unknown base N (N is an unidentified base) content greater than or equal to 10% were removed; (3) Reads without 3 ‘junction sequence were removed; (4) Cut off the 3 ‘joint sequence; (5) Remove sequences shorter than 15 or longer than 35 nucleotides. Then Bowtie tools soft was used, and the clean reads respectively with Silva database, GtRNAdb database, Rfam database and Repbase database sequence alignment. The ribosomal RNA (rRNA), transfer RNA (tRNA), small nuclear RNA (snRNA), small nucleolar RNA (snoRNA) and other ncRNA and repeats were filtered. Finally, the remaining reads were used to detect known miRNA and new miRNA predicted by comparing with known miRNAs from miRbase and Human Genome (GRCh38), respectively. The transcripts per million (TPM) was calculated by comparing the number of read for each miRNA.

### 2.5 Analysis and screening of reference miRNAs and differential miRNAs

Referring to the screening conditions in the reported reference gene validation studies and combined with the actual data of this study, the final screening conditions are determined as follows (34–37): the intra-group mean value of the coefficient of variation (CV) ≤ 20%, and the Log10 (counts)>=4. Besides, platelet and exosome differential miRNA analysis was performed by using DESeq2 R package. The screening criteria were|log2Foldchange| ≥ 0.5, *P* value < 0.05. The platelet differential miRNAs we selected were compared with platelet differential miRNAs that have been studied and other LAC miRNA databases, including TGGA, EMTAB8026, GSE55993, GSE68951, GSE40738, GSE137140. All data analysis was performed by using the statistical programming language R (R Foundation for Statistical Computing, http://www.Rproject.org).

### 2.6 Platelet miRNA isolation, cDNA synthesis and qRT-PCR

A miRNA rapid extraction kit for blood liquid sample (centrifugal column type) (Bioteke, China) were used to isolate platelet miRNA. The assessments of the concentration and quality of miRNA were carried out by Thermo Scientific NanoDrop 2000 Spectrophotometer (Thermo Scientific, USA). Reverse transcription was carried out using a Mir-X™ miRNA First Strand Synthesis Kit (TaKaRa Bio, Dalian, China), following the manufacturer’s instructions. The 10μl system was selected for reverse transcription and the specific system we performed in this study as shown in **Supplementary Table 2**. The design of MiRNA primers used poly (A) tailing-based method. Only forward primer needs to be designed. The reverse primer of miRNA is the general reverse primer provided in the Mir-X™ miRNA First Strand Synthesis Kit.

The primers were designed and synthesized by Tsingke Biological Technology (Beijing, China). All the primers that we used were listed as **Supplementary Table 3**. Quantitative real-time polymerase chain reaction (RT-qPCR) was carried out using the ABI 7500 Real-Time PCR Detection System (Thermo Scientific; USA), in which the amplification and detection steps were combined. All the assays were performed using three biological replicates. A single qPCR reaction was performed in a 20 µL volume containing 10 µL TB Green Premix Ex Taq II(2×), 0.8 µL of each primer, 2 µL of cDNA sample, 0.4 µL ROX Reference Dye II and 6.0 µL water free of RNase and DNase. The melting curve of the qRT-PCR product, the specificity of the primer is better when the melting curve shows a single peak and the miRNAs with a cycle threshold (Ct) value less than 35 in the panel were included in the data analysis.

### 2.7 The analysis of stability and diagnostic value for platelet miRNAs

The average expression stability of the candidate reference miRNAs was evaluated by the comparative Delta CT method (38), BestKeeper (39), and geNorm (40), and NormFinder (41). The differential expression levels of miRNAs in platelets were used t-test or nonparametric tests, and *P value* < 0.05 was considered be statistically significant. The diagnostic value was evaluated by ROC analysis. The comparison of diagnostic performance between platelet differential miRNA and other biomarker and clinical models used ROC and Net Reclassification Index (NRI). All data analysis was performed by using the statistical programming language R (R Foundation for Statistical Computing, http://www.Rproject.org) and the Graphpad Prism 9.3 statistical software.

### 2.8 Target gene prediction

Data from the TGGA database were used to analyze the relationship between differential miRNA and clinical parameters of cancer patients. The target gene prediction of differentially expressed miRNA was implemented by the multiMiR R packages (42). The cellular component (CC), molecular function (MF), and biological process (BP) of target genes by Gene Ontology database (http://geneontology.org). Co-expression of LAC analysis for differential miRNA and target gene was performed via the web of STARBASE (https://starbase.sysu.edu.cn/). The survival analysis of the target gene was analyzed via GEPIA (http://gepia.cancer-pku.cn/index.html).

## 3 Results

### 3.1 Comparison of miRNAs profile in platelets and exosomes

**Figure 2:**
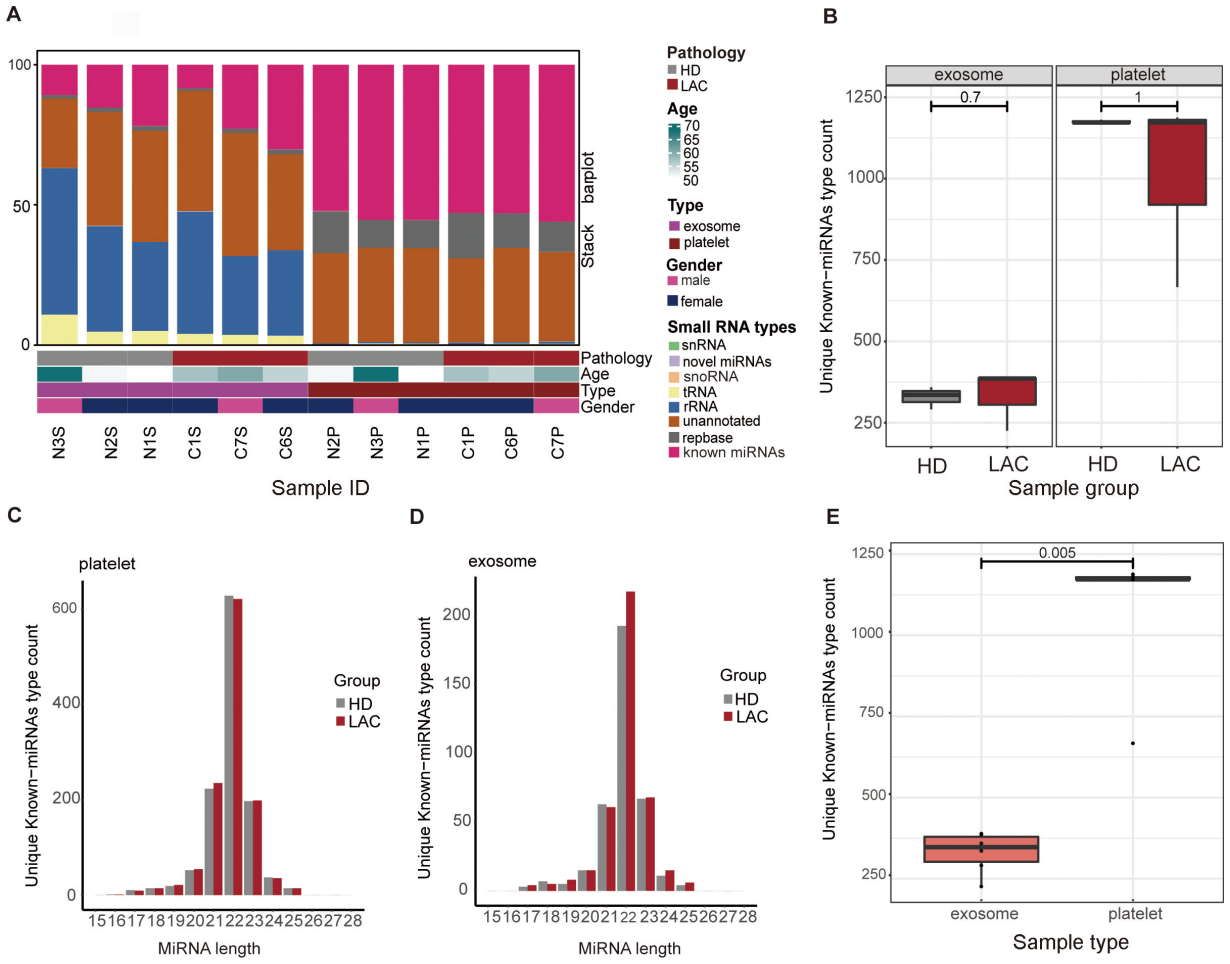
Overview of basic information of platelets and exosomes sequencing. **(A)** Differences in the distribution of small RNA types between platelets and exosomes. **(B)** Differences of unique known-miRNAs type count in platelets (*P=1*) and exosomes(*P=0.7*) between HD group and LAC group. **(C)** Differences of unique known-miRNAs type count between platelets and exosomes. **(D)** Length distribution of platelets and exosomes known miRNAs. **(E)** Differences of unique known-miRNAs type count between platelets and exosomes in our RNA-sequencing data *(P=0.005)*.

Platelets and exosomes RNA-seq results from HD (n=3) and LAC (n=3) showed that miRNAs in platelets and exosomes have significant differences (**Figure 2**). Detailed clinical features from participants are provided in **Supplementary Table 4**. Firstly, the results showed that rRNA accounted for the largest proportion of small RNA species in exosomes, while miRNA accounted for the largest proportion of small RNA species in platelets (**Figure 2A**). There was no difference in the unique known-miRNAs counts in platelets and exosomes detected by RNA-seq examination from HD and LAC groups (*p>*0.05, **Figure 2B**). Besides, the results showed that miRNAs fragment length detected in both exosomes and platelets was mainly 22nt (**Figures 2C, D**), which the length of miRNA was consistent with the current research. Finally, platelet have more he unique known miRNAs counts than exosomes and there are significant differences in the unique known miRNAs counts between platelets and exosome groups (*p*=0.005, **Figure 2E**).

### 3.2 Reference miRNA in platelet for LAC

**Figure 3:**
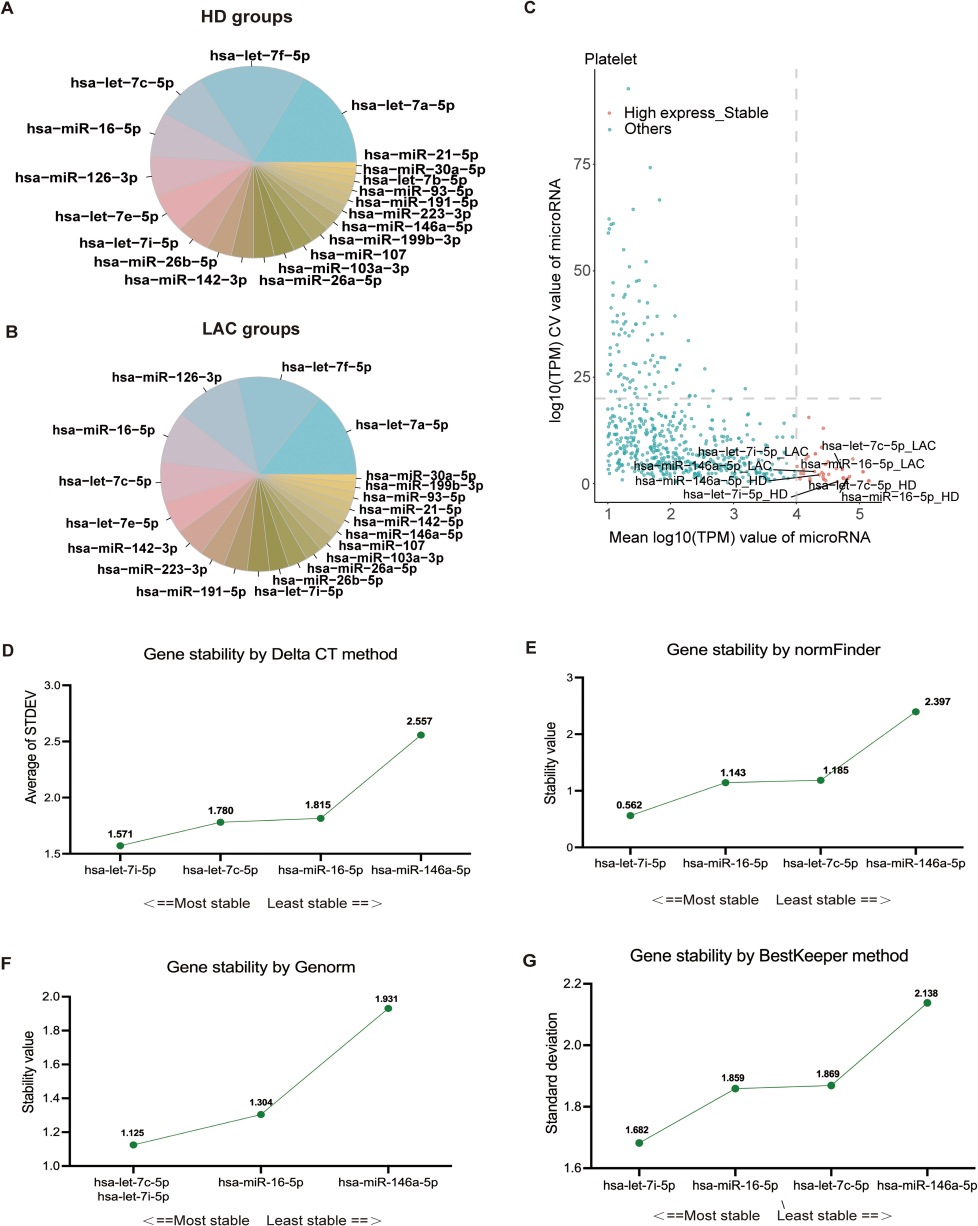
Screening and verification of platelet reference miRNAs. **(A)** The 20 miRNAs with the highest expression levels in healthy donors (HD). **(B)** The 20 miRNAs with the highest expression levels in lupus anticoagulant (LAC) groups. **(C)** Identification of four candidate reference miRNAs in platelets. **(D)** Ranking of the four reference miRNAs based on their expression stability, calculated using Delta CT values (hsa-let-7i-5p > hsa-let-7c-5p > has-miR-16-5p > has-miR-146a-5). **(E)** Stability values for each of the four candidate reference miRNAs obtained from NormFinder analysis (hsa-let-7i-5p > has-miR-16-5p > hsa-let-7c-5p > has-miR-146a-5). **(F)** GeNorm analysis of the four candidate reference miRNAs, indicating stability rankings (hsa-let-7i-5p = hsa-let-7c-5p > has-miR-16-5p > has-miR-146a-5). **(G)** BestKeeper algorithm analysis for assessing the stability of the four candidate reference miRNAs (hsa-let-7i-5p > has-miR-16-5p > hsa-let-7c-5p > has-miR-146a-5). Lower values indicate more stable expression for the normalization factor.

Based on platelet RNA-seq results, we identified the top 20 miRNAs with the highest expression levels in HD and LAC groups (**Figures 3A, B**). According to the screening condition, we selected four stable and highly expressed candidate reference miRNAs (hsa-miR-146a-5p, hsa-miR-16a-5p, hsa-let-7c-5p, and hsa-let-7i-5p) in platelets for further stability verification All four candidate miRNAs were among the top 20 miRNAs with the highest expression levels in both HD and LAC group (**Figure 3C**).

RT-qPCR was used to confirm the expression levels of these four genes in LAC patients (n=16) and HDs (n=17). The baseline characteristics of all participants are listed in **Table 1**. The mean of Ct values of the four reference miRNAs in the 33 subjects are shown in **Supplementary Table 5**. The comparative delta CT algorithm calculated δCt by comparing the relative expression of “miRNA pairs” in each sample, used as a criterion for screening reference genes. As determined by Delta CT method analyses, the-let-7i-5p was the most stable candidate reference miRNA (SD=1.571) (**Figure 3D**).

**Table 1:** Baseline characteristics of all enrolled subjects for validation of candidate reference miRNAs.

Characteristics	Total	HD	LAC
Sex			
male	15 (100%)	10 (66.67%)	5 (33.34%)
female	18 (100%)	7 (38.89%)	11 (61.11%)
Age group, n (%)			
≤ 55years	18 (100%)	10 (55.56%)	8 (44.44%)
> 55 and ≤ 80 years	15 (100%)	7 (46.67%)	8 (53.33%)
Average age	54.79 ± 8.55	54.18 ± 9.29	55.44 ± 7.14
Stage (n)			
I-II			7 (43.75%)
III-IV			6 (37.50%)
NA			3 (18.75%)

HD, healthy donors; LAC, lung adenocarcinoma.

The geNorm is a popular algorithm to determine the most stable reference genes from a set of tested candidate reference genes in each sample panel. The analysis of the GeNorm algorithm found that hsa-let-7c-5p was highly expressed and stable along with hsa-let-7i-5p (**Figure 3E**). In addition, the results of the NormFinder algorithm and the BestKeeper algorithm both showed that the most stable expression of four candidate reference miRNAs was hsa-let-7i-5p (**Figures 3F, G**). Finally, the candidate reference miRNAs were ranked in increasing order of their stability values, and platelet has-let-7i-5p was the best reference miRNA in platelets for LAC (**Table 2**).

**Table 2:** Ranking of the three reference miRNAs stability.

Methods	Reference genes stability value rank			
	First	Second	Third	Fourth
Delta CT	hsa-let-7i-5p	hsa-let-7c-5p	hsa-miR-16-5p	hsa-miR-146a-5p
Bestkeeper	hsa-let-7i-5p	hsa-miR-16-5p	hsa-let-7c-5p	hsa-miR-146a-5p
Normfinder	hsa-let-7i-5p	hsa-miR-16-5p	hsa-let-7c-5p	hsa-miR-146a-5p
Genorm	hsa-let-7i-5p/ hsa-let-7c-5p	hsa-miR-16-5p	hsa-miR-146a-5p	/
Recommended comprehensive ranking	**hsa-let-7i-5p**	**hsa-let-7c-5p**	**hsa-miR-16-5p**	**hsa-miR-146a-5p**

The bold font represents the final staçbility ranking.

### 3.3 Differential miRNAs in platelets from LAC compared with HD

**Figure 4:**
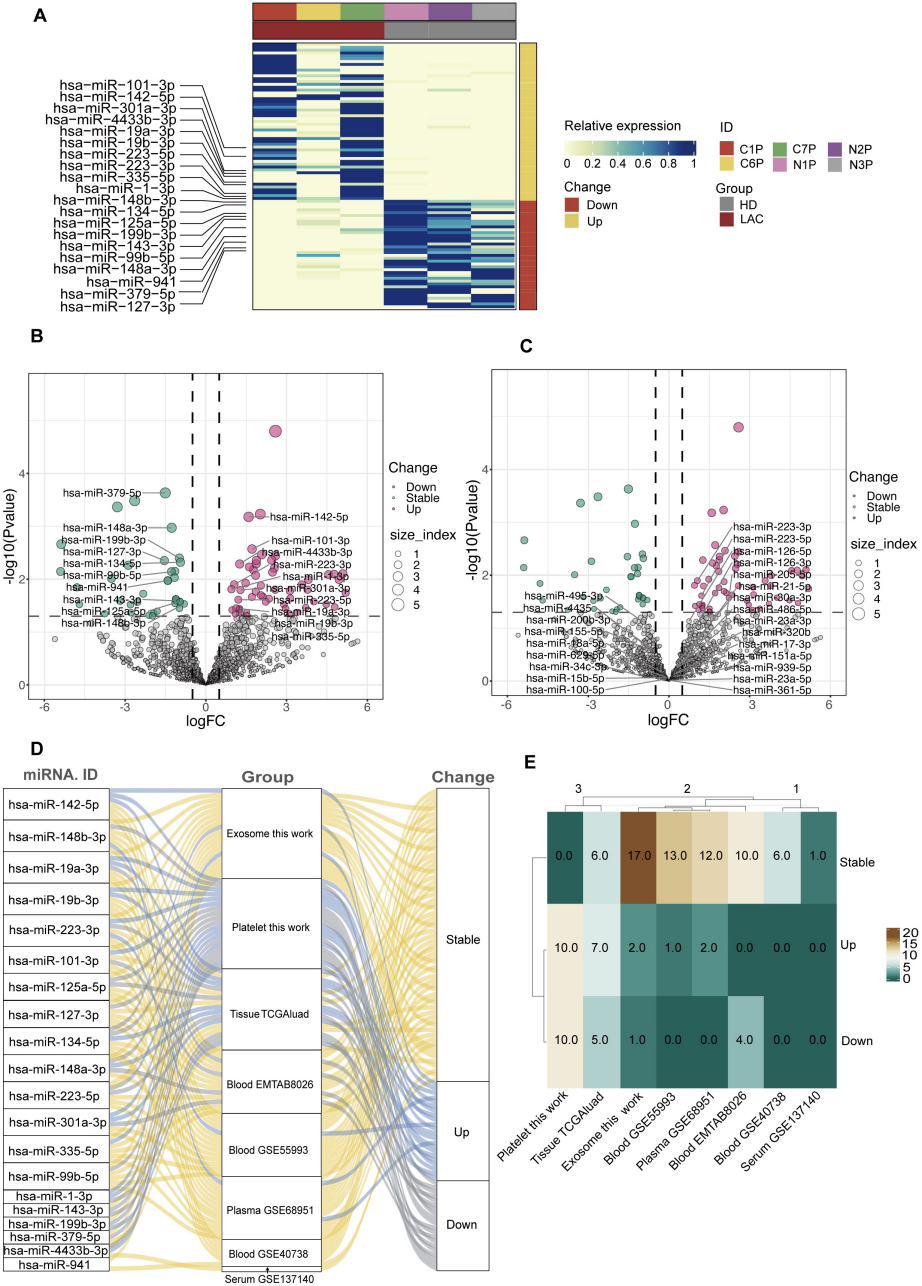
Screening and analysis of platelet differential miRNAs. **(A, B)** The top 10 up-regulated and the top 10 down-regulated differential miRNAs in LAC platelets. **(C)** The platelet differential miRNAs that have been studied and published compare with the 20 differential miRNAs screened by our sequencing data. **(D, E)** The expression and differences of 20 platelet differential miRNAs in other types of sample dataset (TGGAluad, EMTAB8026, GSE55993, GSE68951, GSE40738, GSE137140).

According to the screening criteria, we finally obtained 55 up-regulated miRNAs and 34 down-regulated miRNAs in LAC platelets. In our study, the 10 highest expression up-regulated miRNAs were hsa-miR-223-3p, hsa-miR-142-5p, hsa-miR-1-3p, hsa-miR-101-3p, hsa-miR-223-5p, hsa-miR-335-5p, hsa-miR-19a-3p, hsa-miR-19b-3p, hsa-miR-301a-3p, hsa-miR-4433b-3p, and the 10 highest expression down-regulated hsa-miR-199b-3p, hsa-miR-148b-3p, hsa-miR-148a-3p, hsa-miR-125a-5p, hsa-miR-143-3p, hsa-miR-99b-5p, hsa-miR-379-5p, hsa-miR-134-5p, hsa-miR-127-3p, hsa-miR-941. (**Figures 4A, B**). The heatmap showed that the 20 platelet differential miRNAs could clearly distinguish LAC patients from healthy individuals (**Figure 4A**).

According to the platelet differential miRNAs that have been published, hsa-miR-223-3p and hsa-miR-223-5p were replicated in our results, and this result further demonstrated the reliability of our sequencing data (**Figure 4C**).

In addition, we compared the 20 platelet differential miRNAs we selected with other LAC miRNA datasets. The results showed that most of the miRNAs differentially expressed in platelets were stably expressed in plasma, exosomes, and blood (**Figures 4D, E**). However, of the 20 miRNAs differentially expressed in platelets, 13 were also differentially expressed in tissues (**Figure 4E**). Most of the differentially expressed miRNAs in platelets were also differentially expressed in tissue further demonstrated the potential of platelet miRNAs as a biomarker for liquid biopsy.

### 3.4 Preliminary validation of two differential miRNAs by RT-qPCR assay

**Figure 5:**
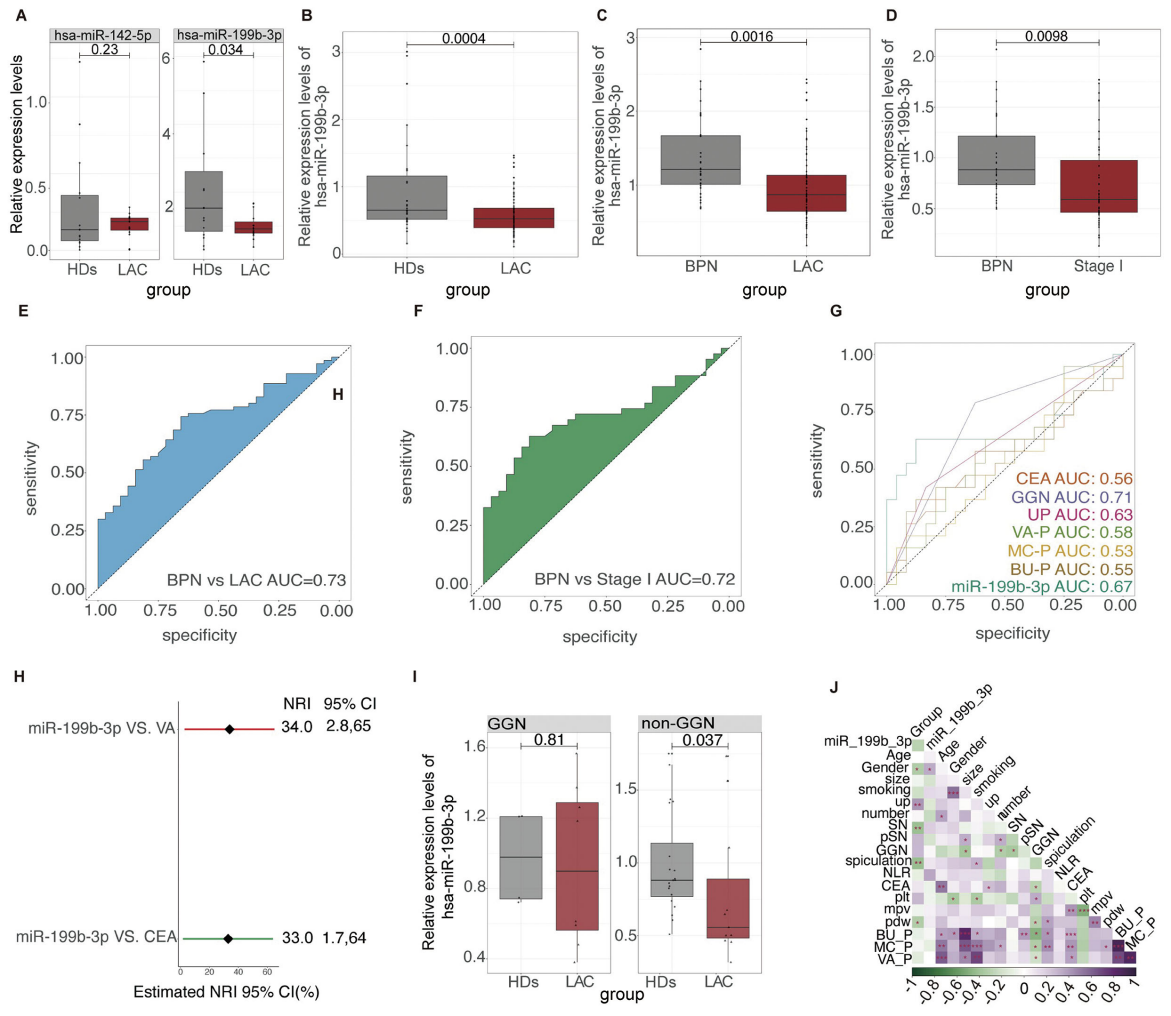
The relative expression level and ROC results of platelet hsa-miR-199b-3p in LAC, HD and BPN groups **(A)** The relative expression levels of platelet hsa-miR-142-5p (*P=0.23*) and platelet hsa-miR-199b-3p between LAC and HD *(P=0.034*). **(B–D)** The relative levels of platelet hsa-miR-199b-3p in between LAC and HD (*P=0.0004)*, LAC and BPN *(P=0.0016)*, and between LAC stage I and BPN *(P=0.0098)*. **(E, F)** The ability of platelet hsa-miR-199b-3p for diagnosis of benign and malignant pulmonary nodules (AUC=0.73) and diagnosis and distinguish of LAC stage I and BPN (AUC=0.72). **(G)** The performance comparison of platelet hsa-miR-199b-3p compared with other clinical models and biomarkers for diagnosis of benign and malignant pulmonary nodules. **(H)** Integrated discrimination improvement for NRI in the diagnosis of benign and malignant pulmonary nodules for hsa-miR-199b-3p and VA models and CEA. **(I)** In GGN and non-GGN, the relative expression of hsa-miR-199b-3p in patients with benign and malignant pulmonary nodules (p=0.81, 0.0037 respectively). **(J)** The relationship among multiple factors, biomarkers, and clinical models for diagnosis of benign and malignant pulmonary nodules.

Since low abundance in total RNA samples make miRNA analysis a challenging task (43, 44). In our study, we chose the differential miRNAs with the highest abundance rather than the Fold Change differences for preliminary verification. The candidate differential miRNAs we selected were hsa-miR-199b-3p and hsa-mir-142-5p. According to the exclusion and inclusion criteria, a total of 30 subjects were included in the first validation, including 15 LAC and 15 HD. The baseline characteristics of all participants are listed in **Table 3**. Preliminary RT-qPCR results showed that the relative expression of hsa-miR-199b-3p in HD and LAC platelets was statistically different (p=0.0034), while the relative expression of hsa-mir-142-5p in HD and LAC platelets was not statistically different (*p*=0.23) (**Figure 5A**). Therefore, we finally selected hsa-miR-199b-3p for further large-scale verification.

**Table 3:** Basic clinical characteristics of the verified subjects.

Characteristics	Preliminary verification		Further verification		
	HD (n=15)	LAC (n=15)	HD (n=31)	BPN (n=32)	LAC (n=70)
Sex					
male	9 (60.00%)	7 (46.67%)	15(48.39%)	17 (43.33%)	23 (61.70%)
female	6(40.00%)	8 (53.33%)	16 (51.61%)	15 (46.88%)	47 (67.14%)
Age group, n (%)					
40–60 years	10 (66.67%)	11 (73.33%)	25 (80.64%)	25 (78.12%)	44 (62.86%)
> 60 years	5 (33.33%)	4 (26.67%)	6 (19.36%)	7 (21.88%)	26 (37.14%)
Average age	55.80 ± 8.79	55.66 ± 11.18	53.26 ± 9.02	59.25 ± 11.88	55.9 ± 11.58
Histological type (n)					
Adenocarcinoma		15 (100%)			70 (100%)
Inflammatory Pseudotumor				32 (100%)	
Stage (n)					
I-II		10 (66.67%)			46 (65.71%)
III-IV		5 (33.33%)			24 (34.29%)
Information of platelets					
Platelets, 109/L	181 ± 56	203 ± 54	201 ± 50	175 ± 45	174 ± 48
MPV, fL	11.1 ± 1.9	11.3 ± 1.5	10.3 ± 1.3	11.5 ± 1.5	11.6 ± 1.5
PDW, fL	16.3 ± 0.28	16.3 ± 0.24	16.2 ± 0.27	16.4 ± 0.37	16.4 ± 0.37

HD, healthy donors; BPN, benign pulmonary nodules; LAC, lung adenocarcinoma.

### 3.5 Further validation of platelet hsa-miR-199b-3p by RT-qPCR assay

To further verification, 70 LAC patients and 31 healthy subjects and 32 BPN patients were included in this study to verify whether hsa-miR-199b-3p could be a biomarker for LAC diagnosis (**Table 3** **).** We used RT-qPCR methods to examine hsa-miR-199b-3p expression levels in platelets in HD group, LAC group and BPN group. The results showed that the expression levels of platelet hsa-miR-199b-3p in HD group were significantly higher than in LAC group (*p*=0.0004, **Figure 5B**). In addition, we also compared the expression levels of hsa-miR-199b-3p in platelets between BPN group and LAC group by using RT-qPCR assay. Surprisingly, the results showed that the expression of hsa-miR-199b-3p was significantly higher in BPN group than in LAC group (*p*=0.0016, **Figure 5C**).

In current clinics, it is very difficult to distinguish LAC stage I with only a single lesion less than 3cm from benign pulmonary nodules. We further evaluated the expression levels of platelet hsa-miR-199b-3p between LAC stage I patients with only a single lesion less than 3cm and BPN patients. To our surprise, the results showed that the expression levels of platelet hsa-miR-199b-3p in LAC stage I group and BPN group was statistically different (*p*=0.0098, **Figure 5D**). The mean of Ct values of platelet has-miR-199b-3p in the 133 subjects were shown in **Supplementary Table 6**. According to the mean value of independent samples of the two groups was compared to ensure that the test performance reached 0.8, and the number of experimental groups and control groups were qualified.

### 3.6 Diagnostic value of platelet hsa-miR-199b-3p

We used ROC curves to analyze the ability of platelet hsa-miR-199b-3p to distinguish benign from malignant pulmonary nodules. The analysis results showed that platelet hsa-miR-199b-3p has good diagnostic accuracy in distinguishing LAC group from BPN group and the AUC for the ROC was 0.73 (95% CI, 0.5574 to 0.7850) (**Figure 5E**), and the AUC for the ROC was 0.72 (95% CI, 0.6285 to 0.8255) in the diagnosis of BPN group and LAC stage I group (**Figure 5F**).

According to the participants with complete clinical information and laboratory information. The performance of hsa-miR-199b-3p to diagnose benign and malignant pulmonary nodules was compared with other clinical models and CEA (**Figure 5G**). The results showed that the AUC of platelet hsa-miR-199b-3p in the LAC was 0.67 (95%CI, 0.4889 to 0.8576), while the AUC of CEA in LAC was 0.55(95%CI, 0.3722 to 0.7352). The diagnostic performance to distinguish benign from malignant pulmonary nodules of other clinical models including VA model (AUC=0.58, 95%CI, 0.4034 to 0.7523), BU model (AUC=0.55, 95%CI, 0.3735 to 0.7361), and MC model (AUC=0.53,95%CI, 0.3499 to 0.7028) also presented limitation. Therefore, platelet hsa-miR-199b-3p have shown significant clinical values by showing a high sensitivity, specificity and accuracy for LAC diagnosis and benign and malignant pulmonary nodules diagnosis. Platelet hsa-miR-199b-3p had a better reclassification ability than the best clinical model (VA model) and CEA with significant NRI (respectively, 0.33, 95%CI, 0.0028-0.65; 0.34, 95%CI, 0.0017, 0.0064) (**Figure 5H**).

Finally, we established a logistic regression model with three important indicators (nodule size, CEA and TEP Has-miR-199b-3p). Then we compared the diagnostic efficiency of the model after removing the three indicators respectively. The diagnostic efficiency of diagnostic model diagnostic efficiency (AUC=0.64), however, when we are removing our indicators (TEP Has-miR-199b-3p), the diagnostic performance of the diagnostic model decreased the most (AUC=0.54) (**Supplementary Figure 2**). Therefore, the TEP Has-miR-199b-3p is an important indicator for the diagnosis model of benign and malignant pulmonary nodules.

However, the method of distinguishing benign and malignant lung nodules according to whether lung nodules are ground glass nodules (GGNs) is better (AUC =0.71) (**Figure 5G**). Then, patients with lung nodules were divided into GGN group and non-GGN group. We found that the expression of platelet hsa-miR-199b-3p in the non-GGN group was statistically different between patients with benign and malignant pulmonary nodules (*p*=0.037) (**Figure 5I**). It is proved that platelet hsa-miR-199b-3p also can further play an essential role in the diagnosis of benign and malignant in non-GNN group. Finally, we also analyzed the relationship between multiple factors. We can find platelet hsa-miR-199b-3p also seem to correlate the gender (**Figure 5J**).

### 3.7 Prediction and analysis of target genes of hsa-miR-199b-3p

**Figure 6:**
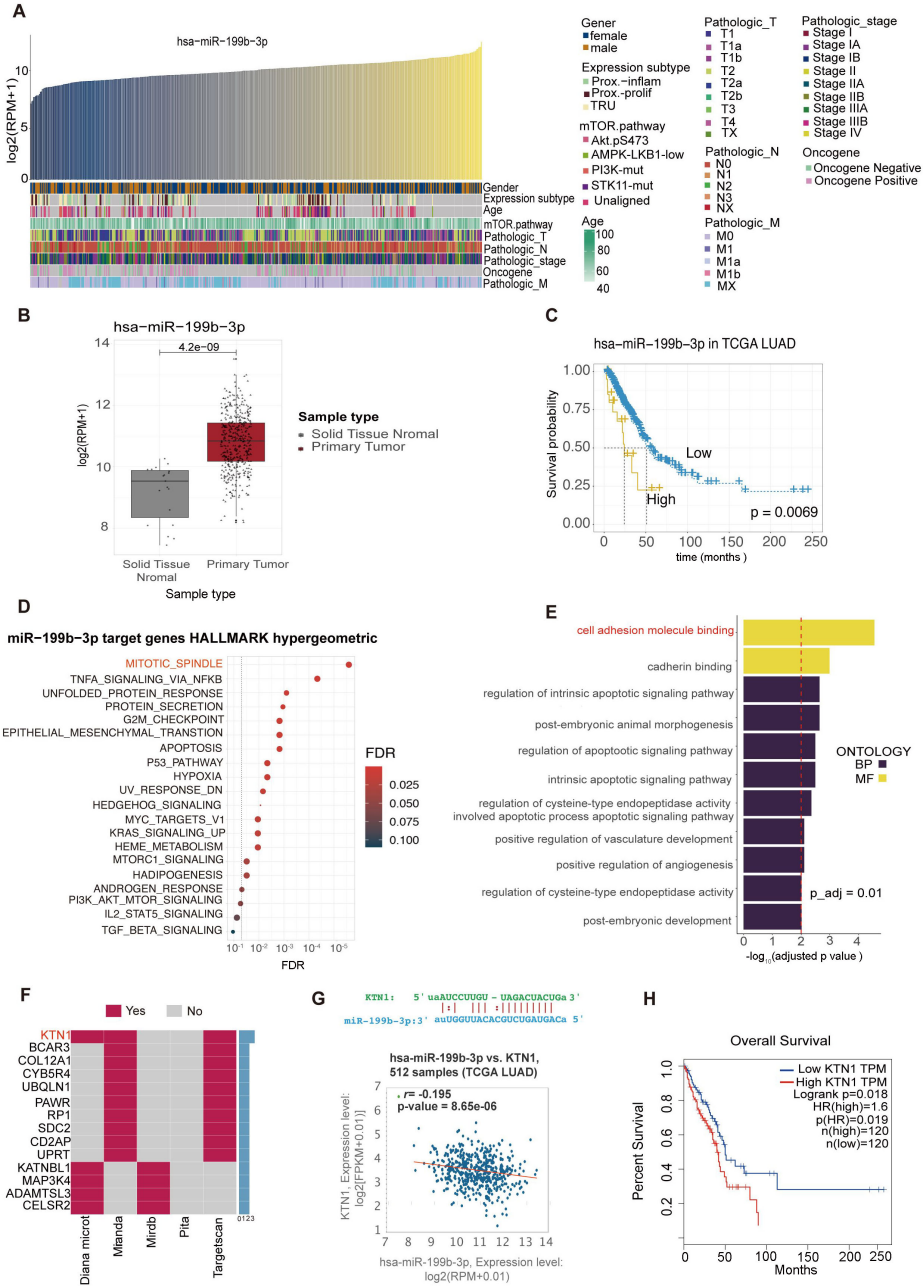
Bioinformatics analysis of hsa-miR-199b-3p and target genes predictions of hsa-miR-199b-3p. **(A)** Correlations between the expression levels of hsa-miR-199b-3p and clinical parameters of LAC cancer subjects. **(B)** The expression levels of hsa-miR-199b-3p in solid tissue normal and primary tumor *(P <0.05)*. **(C)** The association of hsa-miR-199b-3p expression and survival probability *(P=0.0069).***(D, E)** GO term enrichments analysis of target genes of hsa-miR-199b-3p. here we listed the top 11GO terms *(all P < 0.05)* belonging to Biological Process, Molecular Function categories, respectively. **(F)** The same target genes predicted by at least two of the five prediction methods (KTN1 gene appeared in three prediction methods among total five prediction methods). **(G)** The complementary sequence of hsa-miR-199b-3p and the KTN1 gene and the correlation of co-expression between the KTN1gene and hsa-miR-199b-3p in lung adenocarcinoma (r=0.195, *P=8.56e-06*). **(H)** The association of KTN1 gene expression with survival probability in LAC cancer (p=0.0019).

Correlations between the expression levels of hsa-miR-199b-3p and clinical parameters of LAC cancer subjects shown in **Figures 6A–C**. The expression of hsa-miR-199b-3p at different ages, genders, and stages of cancer will help further study the potential significance of hsa-miR-199b-3p as a cancer marker (**Figure 6**). We can find that the expression levels of hsa-miR-199b-3p in solid tissue normal and primary tumor was statistically different (*p <*0.05) (**Figure 6B**). Further evidence of the potential significance of hsa-miR-199b-3p as a biomarker for the diagnosis of LAC. (All data comes from TCGA database). Besides, the high expression of hsa-miR-199b-3p was associated with low survival probability (*p*=0.0063) (**Figure 6C** **).**

The target gene prediction of hsa-miR-199b-3p was implemented by the multiMiR R packages. According to five different target gene prediction methods (Diana Micro, ELMMO, Miranda, Pita, Targetscan), 2593 possible target genes of hsa-miR-199b-3p were obtained. Then we integrated the information of the two databases (Mirtarbase, Tarbase), and found that 402 of the 2593 predicted target genes have been validated. We selected the top 10% predicted genes of each method for gene enrichment analysis, and the results showed that the main functions of target genes of hsa-miR-199b-3p were participated in mitotic spindle and cell adhesion molecule binding (**Figures 6D, E**). In addition, based on the five prediction methods, we found that the KTN1 gene appeared in all three prediction methods (**Figure 6F**).

By retrieving UALAN database (https://ualcan.path.uab.edu/index.html), we found that *KTN1* expression was higher in cancer tissues than in para-cancer tissues (**Supplementary Figure 3**). The complementary sequence of hsa-miR-199b-3p and the KTN1 gene shown in **Figure 6G**. Besides, in lung adenocarcinoma, there was a significant correlation of co-expression between the *KTN1*gene and hsa-miR-199b-3p (r=0.195, p=8.56e-06) (**Figure 6G**). Finally, Higher the *KTN1* gene expression was associated with lower survival in LAC cancer, consistent with hsa-miR-199b-3p results (**Figure 6H**). Therefore, we thought that the *KTN1*gene was the most likely target gene for RNA hsa-miR-199b-3p. Then we conducted some preliminary verification on the gene expression of *KTN1* in platelets of LAC and healthy donor (**Supplementary Figure 4**). It can be preliminarily found that there are statistical differences in the expression of TEPs *KTN1*(P<0.0001). We found an interesting point that the expression of *KTN1* in TEPs is opposite to the expression in tumor cells and tissues. Our team suspected that it may be consistent with the current mechanism hypothesis of TEPs. Mechanism hypothesis of TEPs formation is that platelets educate tumor cells by highly efficient transference of lipids, proteins and RNA through different mechanisms (28).

## 4 Discussion

We conducted miRNA sequencing of platelets and plasma exosomes to investigate miRNA expression differences and their relationship with lung adenocarcinoma (LAC). Differentially expressed platelet miRNAs were identified and compared with LAC-related miRNA profiles from other sources to assess their potential as liquid biopsy biomarkers. We then validated selected platelet miRNAs using RT-PCR in a cohort of 71 LAC patients, 31 healthy donors, and 32 patients with benign pulmonary nodules. Key findings of our study are discussed below.

Contextualizing our platelet miRNA findings within the broader immunobiology of cancer, platelets emerge as pivotal immunomodulators in the tumor microenvironment (TME) (45). While our study focuses on LAC diagnostic biomarkers, it is noteworthy that platelets exhibit dual roles across malignancies: In colorectal cancer, Erbin-deficient platelets transfer mitochondrial metabolites to B cells, enhancing PD1 degradation and antitumor immunity (46); conversely, in triple-negative breast cancer (TNBC), procoagulant platelets deliver immune checkpoint (IC) molecules that dysregulate leukocytes to drive immune escape (47). Critically, such immunomodulatory plasticity may operate in LAC, where platelet-derived miRNAs (like miR-199b-3p) could similarly regulate TME interactions – a hypothesis warranting mechanistic validation.

Firstly, we proved that the expression level of platelet hsa-miR-199b-3p was significantly difference in LAC patients as compared to healthy person (*p*=0.0004). Lung cancer is one of the leading causes of death, with an increasing incidence and has long been a disease characterized by advanced diagnosis (48). LAC is the most frequent subtype of lung cancer. Therefore, early diagnosis of LAC is very important. With the accumulation of molecular knowledge gained from emerging technology platforms (such as next-generation sequencing and other omics platforms), lung cancer screening, and personalized therapy has been made much progress in recent years (49–51). As reported by the national lung cancer screening guidelines in China and Canada, biomarker detection was also an area of continued concern (30, 52). Tumor biomarkers contribute to the early diagnosis of lung cancer and play important roles in treatment, monitoring, and prognosis prediction (53). Previous studies have demonstrated the potential of circulating tumor cells (CTCs), cell-free circulating tumor DNA (ctDNA), exosomes, and platelets as biomarkers for lung cancer diagnosis (25, 53–55). However, platelet miRNAs can be potential biologic diagnostic markers for LAC have not been validated. Our results certified that platelet hsa-miR-199b-3p can be a potential biomarker for diagnosing the LAC.

A limitation of this study is the incomplete elucidation of the biological mechanism by which platelet hsa-miR-199b-3p regulates the occurrence and development of lung adenocarcinoma (LAC). However, previous research has suggested that miR-199a-3p/5p and miR-199b-3p may function as tumor suppressors in non-small cell lung cancer (NSCLC) by inhibiting the mTOR signaling pathway through targeting Rheb (56). Another study proved that the up-regulation of miR-199a-3p significantly inhibited NSCLC growth *in vivo*, and reduced A549 cell proliferation and promoted mitochondrial-mediated apoptosis, through down-regulation of Zinc Finger E-box Binding Homeobox 1 (*ZEB1)* (57). Additionally, miR-199a-3p has been reported to play an anti-tumorigenic role in LAC by targeting anterior gradient 2 (*AGR2*) (58). MiR-199a-3p and miR199b-3p are identical in sequence and publications are interchangeable (59, 60).

Emerging evidence implicates platelet-derived miR-199b-3p as a key regulator of LAC progression through targeting kinectin 1 (KTN1). Although no prior studies have directly linked this miRNA-mRNA pair, our computational analysis using a highly cited prediction tool identified KTN1 as the top candidate target (42), supported by conserved binding sites in its 3’UTR and reciprocal expression patterns in LAC (↓miR-199b-3p and ↑KTN1; *p<0.0001*). Critically, KTN1 mRNA is present in platelets and significantly downregulated in LAC patients versus healthy donors—a finding validated by both our RT-qPCR data (*P<0.0001*) and independent platelet transcriptome databases (PEA, PltDB). This platelet-specific dysregulation contrasts with KTN1’s pan-cancer oncogenic role: Tumor tissue analyses (UALCAN) demonstrate consistent KTN1 upregulation across malignancies, where high expression correlates with poor survival in LAC, HCC, and TNBC through driving metastasis, NF-κB-mediated CXCL8 activation, and immune evasion (61).

The platelet miR-199b-3p/KTN1 axis may thus represent a novel liquid biomarker-driven pathway in LAC pathogenesis. Mechanistically, reduced platelet miR-199b-3p in LAC likely derepresses KTN1, mirroring its established tumor-promoting functions: CRISPR/Cas9 studies confirm KTN1 knockout suppresses proliferation, inhibits migration/invasion, and dysregulates innate immune responses in HCC/TNBC models (62, 63). This paradigm aligns with our observed inverse correlation between miR-199b-3p and KTN1 in LAC cohorts (r=-0.195, p=8.56e-06) and their concordant survival impact (low miR-199b-3p + high KTN1 → mortality risk; *p<0.001*). While direct targeting validation is ongoing, the convergence of computational prediction, platelet-specific expression changes, and functional oncology evidence positions this axis as a promising therapeutic target—particularly given platelet miRNA’s accessibility for liquid biopsy-based interception of KTN1-driven oncogenesis.

Besides, our study demonstrated a significant difference in platelet hsa-miR-199b-3p expression levels between lung adenocarcinoma (LAC) patients and those with benign pulmonary nodules (p=0.0016). This finding is particularly relevant given that approximately 95% of all identified pulmonary nodules are benign. highlighting the potential of platelet hsa-miR-199b-3p as a biomarker for distinguishing LAC from benign nodules (9). Correctly distinguishing between benign and malignant pulmonary nodules can help early diagnosis of lung cancer patients and avoid overtreatment of benign nodules (9, 64). At present, the diagnosis of benign and malignant pulmonary nodules is mainly based on tissue biopsy. Because tissue biopsy is the invasive procedure, An accurate noninvasive test is needed to identify malignant nodules and reduce unnecessary invasive tests (65). However, many biomarkers of liquid biopsy can only distinguish healthy person from lung cancer and have certain limitations in the diagnosis of benign and malignant lung nodules. For example, a prospective study evaluating CTCs in 150 patients with a suspicious or a diagnosis of primary lung cancer. The results showed that CTC count was numerically higher in lung cancer patients compared to patients with non-malignant disease, but the ROC curve did not disclose a good discrimination (66). In our study, the ROC curve of platelet hsa-miR-199b-3p did a good discrimination between LAC patients and patients with benign pulmonary nodules (AUC=0.73). Besides, compared with other clinical models (VA model, BU model, MC model) and biomarker (CEA) for the diagnosis of benign and malignant lung nodules, platelet hsa-miR-199b-3p have shown more significant clinical values by showing a higher sensitivity, specificity and accuracy for LAC diagnosis and benign and malignant pulmonary nodules diagnosis.

Most importantly, current diagnostic methods face significant challenges in differentiating between benign nodules and early-stage lung cancer, particularly stage I tumors with nodules smaller than 3cm in diameter (64, 65). Our results showed that there was also a significant difference in expression of platelet hsa-miR-199b-3p between LAC (stage I) and benign nodules. Our study also proved that platelet hsa-miR-199b-3p have significant clinical values for distinguishing LAC stage I and benign pulmonary nodules (AUC=0.72). Therefore, platelet hsa-miR-199b-3p has a good diagnostic value not only in the early detection of lung adenocarcinoma, but also in the differentiation of benign and malignant lung nodules. Finally, we predicted the possible target genes of hsa-miR-199b-3p and analyzed the biological processes that might be involved in it. It laid a foundation for the subsequent mechanism research on platelet hsa-miR-199b-3p regulation of the carcinogenesis, development, and prognosis of LAC.

Secondly, with the past few years, more than 1000 human miRNAs have known (67). The miRNA expression levels can be correlated with cancer type, stage, and other clinical variables, so miRNA profiling can be used as a tool for cancer diagnosis and prognosis (68, 69). An understanding of exosomes is continuously evolving because of robust laboratory-based research (55). Exosome miRNAs are now accepted as potential biomarkers in many diseases, including cancer (18). However, there are certain limitations of exosome extraction methods are summarized in a study. Exosomes extraction is time-consuming and costly (70). On the contrary, platelets as an emerging biological source of liquid biopsy which has several advantages, including their abundance, easy isolation, relatively higher RNA quality, and their ability to respond to external signals to process RNA (18). Meanwhile, platelet miRNAs expression is also widely concerned as biomarkers for a variety of diseases. There has been a growing body of evidence to reveal the roles of platelet miRNAs in various pathophysiological processes as well as cancer pathogenesis, such as platelet miR-34c-3p and miR-18a-5p as potential liquid biopsy biomarkers for nasopharyngeal carcinoma diagnosis and miRNA-223 delivered by platelet-derived micro vesicles promotes lung cancer cell invasion via targeting tumor suppressor *EPB41L3* (25, 71).

Our miRNA sequencing results revealed that platelets contain a greater quantity and diversity of miRNAs compared to exosomes. Analysis of various miRNA sequencing datasets showed that the 20 differentially expressed platelet miRNAs we identified were stably expressed in plasma, blood, serum, and exosomes of both healthy individuals and LAC patients. Notably, 13 out of these 20 miRNAs were also differentially expressed in tissue samples. This higher consistency between platelet and tissue differential miRNAs underscores the potential of platelet miRNAs as liquid biopsy biomarkers. These findings collectively suggest that platelet miRNAs may be more suitable as potential biomarkers for liquid biopsies in LAC compared to exosome miRNAs, despite the latter receiving more attention in recent years.

Finally, with the development of microarrays and sequencing in recent years, many high-throughput gene expression data have been accumulated (72). Several conventional reference genes such as glyceraldehyde-3-phosphate dehydrogenase (*GAPDH*) and b-actin (*ACTB*) have varied significantly under certain experimental conditions and for biological conditions, such as type, evolution, differentiation, development and treatment of cancer, and other diseases or for comparing different physiological stages of a single organ (73–75). Besides, many commonly used reference miRNAs have also been found to be unstable, such as U6, RNU6B, and RNU48 in different cancers (73, 76, 77). Reliable reference miRNAs are crucial for accurate quantification of miRNA expression using qRT-PCR (78). However, normalization of platelet miRNA expression remains a significant challenge in biomarker discovery and validation of single miRNA qRT-PCR assays. It is essential to ensure that observed differences in miRNA expression are directly attributable to the disease under study, rather than to other confounding factors.

To validate the expression levels of four candidate platelet reference miRNAs in LAC patients and healthy donors (HDs), we employed RT-qPCR. The stability of these candidates was assessed using multiple internal stability analysis software programs, including the comparative delta-Ct method, geNorm, NormFinder, and BestKeeper. Our analysis identified hsa-let-7i-5p as the most suitable reference miRNA in platelets of LAC patients. This finding provides a standard for quantitative detection of platelet miRNA expression levels using hsa-let-7i-5p as an internal control.

## 5 Conclusion

In conclusion, our findings suggest that platelet miRNAs may be more suitable as potential biomarkers for liquid biopsies in lung adenocarcinoma (LAC) compared to exosome miRNAs, despite the latter receiving more attention in recent years. Specifically, platelet hsa-let-7i-5p emerged as the most suitable reference miRNA in LAC platelets. Moreover, platelet hsa-miR-199b-3p demonstrated potential as an essential biomarker for LAC diagnosis and differentiation between benign and malignant pulmonary nodules.

## Data Availability

The dataset is available under the project ID PRJCA011324. You can access it via the following link: https://ngdc.cncb.ac.cn/search/specific?db=&q=PRJCA011324.
