# Supplementary material for: MiRNA sequencing of platelet and exosome revealed platelet miR-199b-3p as a potential biomarker in lung adenocarcinoma
Source: Front Immunol. 2025 Aug 29;16:1619448. doi: 10.3389/fimmu.2025.1619448 (PMC12427126; doi:10.3389/fimmu.2025.1619448)
Supplement: Supplementary file 1 [file Supplementaryfile1.docx]

# Supplemental Table 1. The primers used for RT-qPCR

| **Primers** | **Sequence (5ʹ~3ʹ)** |
| --- | --- |
| hsa-miR-16-5p | 5’-CGC-GTA-GCA-GCA-CGT-AAA-TAT-3’ |
| hsa-miR-146a-5p | 5’-CCG-CGT-GAG-AAC-TGA-ATT-CCA-3’ |
| hsa-let-7c-5p | 5’-CGC-GTG-AGG-TAG-TAG-GTT-GT-3’ |
| hsa-let-7i-5p | 5’-CGT-GAG-GTA-GTA-GTT-TGT-GCT-G -3’ |
| hsa-miR-142-5p | 5’-CGC-GCG-CAT-AAA-GTA-GAA-AGC-3’ |
| hsa-miR-199b-3p | 5’-CGC-GAC-AGT-AGT-CTG-CAC-AT-3’ |

| ID | Group | gender | age | pathology | stage | T | N | M |
| --- | --- | --- | --- | --- | --- | --- | --- | --- |
| N1S | Normal | Female | 50 |  |  |  |  |  |
| N1P | Normal | Female | 50 |  |  |  |  |  |
| N2S | Normal | Female | 51 |  |  |  |  |  |
| N2P | Normal | Female | 51 |  |  |  |  |  |
| N3S | Normal | Male | 68 |  |  |  |  |  |
| N3P | Normal | Male | 68 |  |  |  |  |  |
| C1S | Cancer | Female | 57 | LACC | ⅠA | 1 | 0 | 0 |
| C1P | Cancer | Female | 57 | LACC | ⅠA | 1 | 0 | 0 |
| C6S | Cancer | Female | 55 | LACC | ⅠA | 1 | 0 | 0 |
| C6P | Cancer | Female | 55 | LACC | ⅠA | 1 | 0 | 0 |
| C7S | Cancer | Male | 60 | LACC | ⅠB | 2 | 0 | 0 |
| C7P | Cancer | Male | 60 | LACC | ⅠB | 2 | 0 | 0 |

# Supplemental Table 2. Characteristics of enrolled subjects for sequence reads analysis

# Supplementary Table 3 The Ct values of the four candidate reference genes in the 33 subjects.

| **Group** | **hsa-let-7c-5p** | **hsa-let-7i-5p** | **hsa-miR-16-5p** | **hsa-miR-146a-5p** |
| --- | --- | --- | --- | --- |
| HC | 25.895 | 26.47 | 21.805 | 24.025 |
| HC | 24.8 | 23.925 | 20.67 | 22.08 |
| HC | 23.18 | 24.615 | 21.335 | 24.1 |
| HC | 23.745 | 25.27 | 21.845 | 18.35 |
| HC | 19.59 | 21.43 | 18.465 | 21.005 |
| HC | 19.51 | 21.105 | 18.27 | 20.98 |
| HC | 19.59 | 21.39 | 18.275 | 20.52 |
| HC | 21.525 | 22.84 | 19.82 | 22.41 |
| HC | 21.095 | 22.49 | 19.95 | 17.455 |
| HC | 27.19 | 25.77 | 22.02 | 18.65 |
| HC | 27.795 | 29.275 | 26.59 | 26.92 |
| HC | 24.12 | 23.485 | 23.63 | 23.195 |
| HC | 19.61 | 21.355 | 18.75 | 20.81 |
| HC | 22.235 | 22.235 | 19.75 | 22.08 |
| HC | 21.045 | 21.045 | 18.745 | 21.35 |
| HC | 20.995 | 20.995 | 18.06 | 20.96 |
| HC | 21.925 | 21.925 | 19.35 | 21.845 |
| LAC | 24.32 | 24.035 | 20.22 | 17.41 |
| LAC | 25.72 | 24.39 | 26.94 | 19.135 |
| LAC | 23.655 | 23.57 | 24.025 | 19.77 |
| LAC | 21.545 | 23.06 | 20.345 | 22.665 |
| LAC | 19.12 | 20.7 | 18.01 | 20.755 |
| LAC | 21.86 | 23.43 | 20.915 | 23.81 |
| LAC | 21.94 | 23.34 | 20.975 | 23.83 |
| LAC | 23.79 | 24.325 | 20.235 | 22.08 |
| LAC | 24.31 | 24.55 | 20.875 | 23.68 |
| LAC | 26.62 | 27.405 | 23.295 | 25.31 |
| LAC | 23.42 | 25.38 | 22.26 | 24.415 |
| LAC | 21.81 | 23.675 | 20.725 | 22.845 |
| LAC | 20.475 | 21.76 | 19.165 | 21.28 |
| LAC | 24.55 | 26.855 | 23.865 | 26.56 |
| LAC | 20.22 | 21.835 | 19.205 | 21.86 |

HC:Healthy control, LAC:Lung adenocarcinoma

**Supplemental Fig 1**

**Supplemental Fig 1**

**
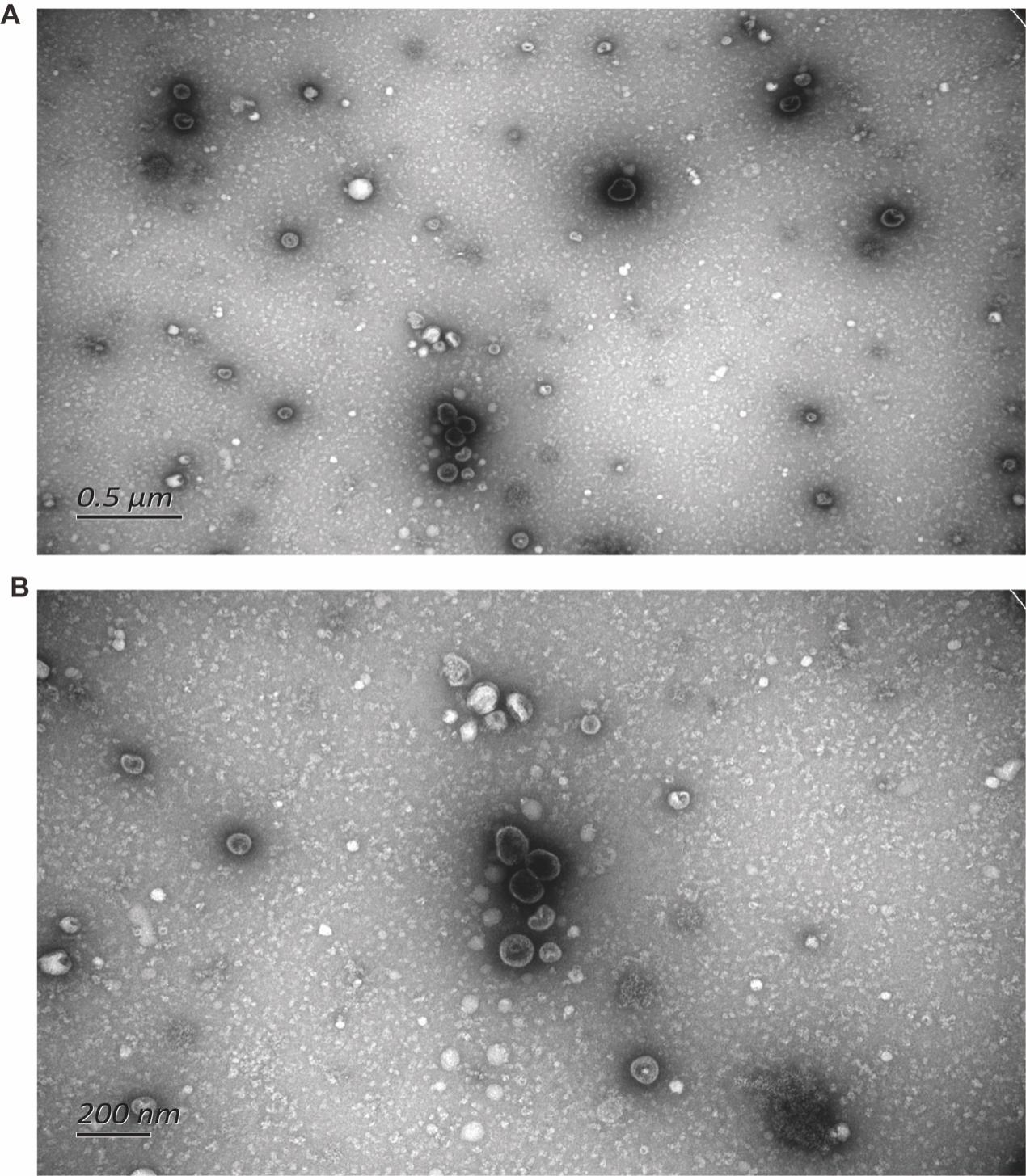
**

Supplemental Fig 1: Electron microscopic view of plasma exosome. A: Electron microscopic view of 0.5um plasma exosome. B: Electron microscopic view of 200nm plasma exosome.

**Supplemental Fig 2**

**
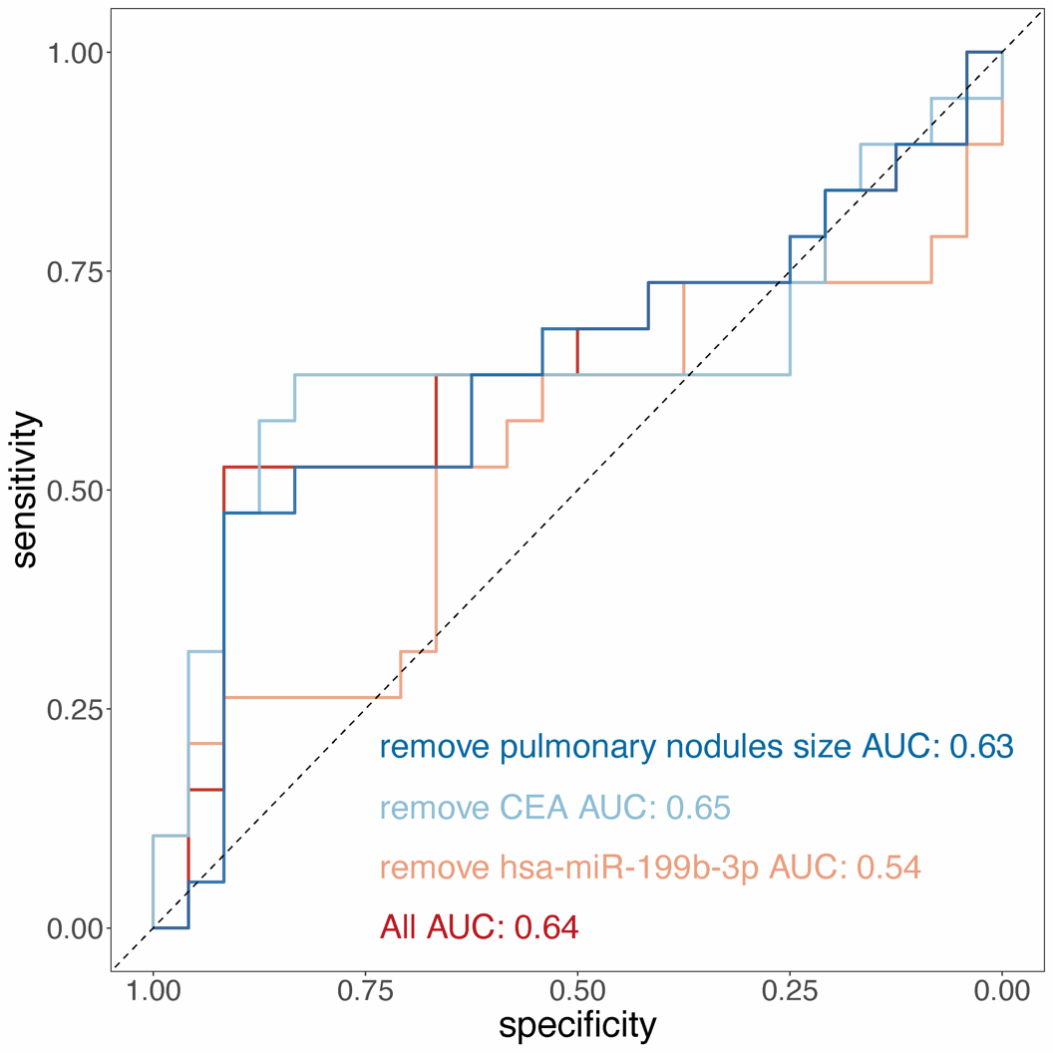
**

Supplemental Fig 2: The logistic regression model for benign and malignant pulmonary nodules diagnosis (size, CEA and TEP-hsa-miR-199b-3p).

**Supplemental Fig 3**


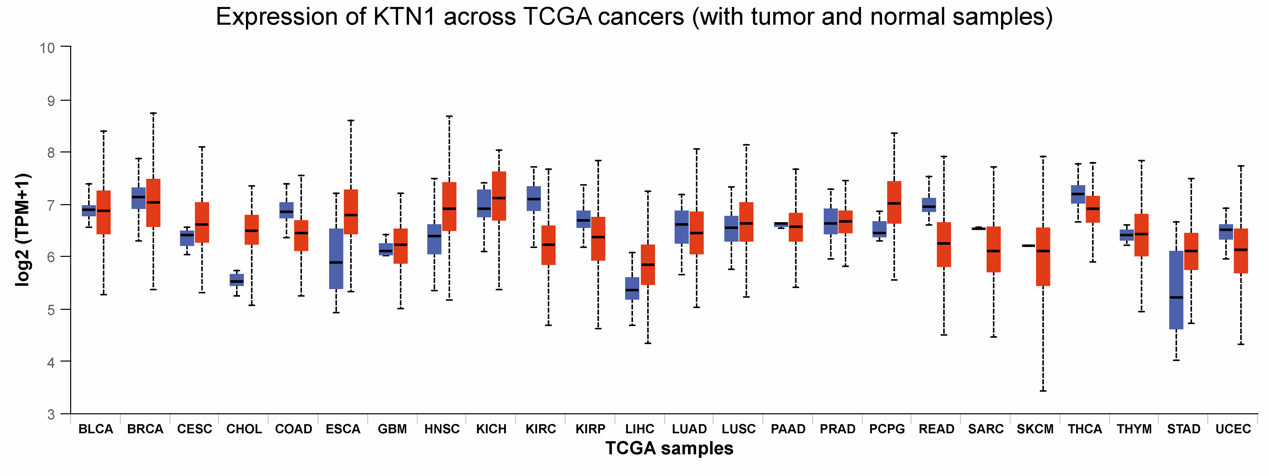


Supplemental Fig 3: The expression of KTN1 across TGGA cancers with tumor (red) and normal (blue) samples

**Supplemental Fig 4**


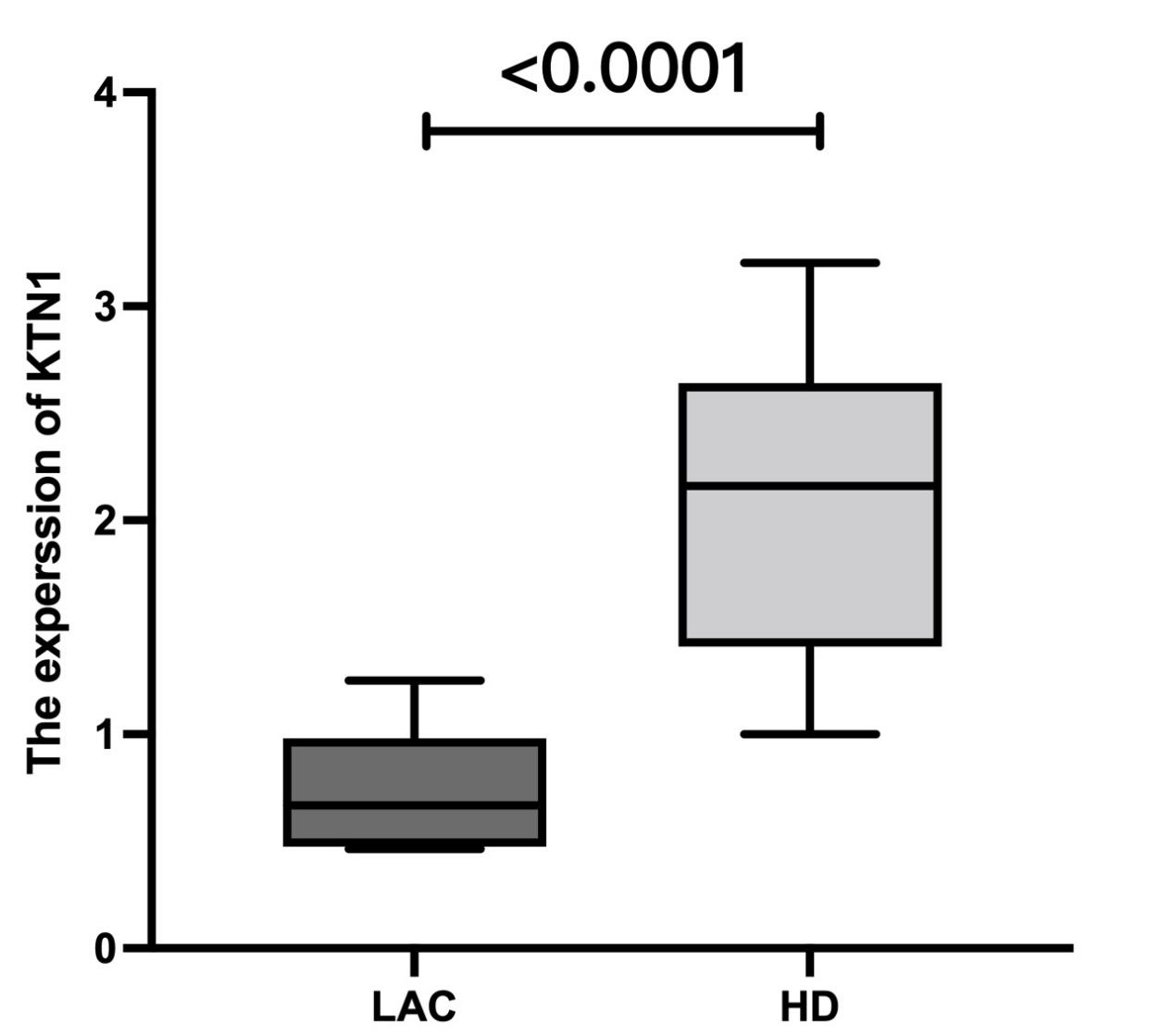


Supplemental Fig 4: The relative expression level of TEPs KTN1 in LAC, and HD (P<0.0001).
